# Swipe now, regret later? How credit cards reduce the appeal of safe choices

**DOI:** 10.3389/fpsyg.2025.1517460

**Published:** 2025-06-04

**Authors:** Hui-Hsi Hung, Yin-Hui Cheng, Shih-Chieh Chuang, Tzu-Ming Wang

**Affiliations:** ^1^Fujian Digital Media Economy Research Center, Fujian Social Science Research Base, Fujian, China; ^2^Department of International Business, National Taichung University of Education, Taichung, Taiwan; ^3^Department of Business Administration, College of Management, National Chung Cheng University, Minhsiung, Chiayi County, Taiwan

**Keywords:** credit card, cash, pain of paying, compromise effect, payment forms

## Abstract

Most research on the influence of decision-making on the compromise effect has focused on paying with cash rather than with a credit card. The experimental investigations of this paper revealed that the compromise effect was reduced when consumers paid with a credit card rather than with cash, and that the pain of paying played a mediating role between the payment form and the occurrence of the compromise effect. In addition, the authors successfully excluded alternative explanations such as differences in price, product category, and attribute importance. Finally, this paper showed that the impact of cash payments on the compromise effect was stronger among tightwads than among spendthrifts.

## Introduction

People face many unusual situations every day and must choose between several options. When making choices, people are likely to select the option with the highest value or utility for them: a principle called value maximization (Simonson and Tversky, 1992; Tversky and Shafir, 1992). Conceptually, value maximization provides an important link between classical economic theory and consumer choice theory, and has been widely applied in theory and practice. The preference invariance hypothesis in value maximization holds that a person’s preferences for a specific set of alternatives is context-independent and that introducing a new option to their choice set will not change their preference hierarchy for existing alternatives (Chuang et al., 2012a; Simonson and Tversky, 1992). For example, when a consumer prefers Brand X over Brand Y in one context (when these two brands are the only ones available), they will not prefer Brand Y over Brand X in another context (when a third option, Brand Z, is added to the original choice set). In such cases, each option has its value, and when one option is better than the others in all respects, it is easy for people to quickly make a final choice: a clear illustration of the value maximization principle.

However, studies have shown that in real-life situations, people’s choices are more complicated and context-dependent. In particular, in cases where each alternative in the choice set has both advantages and disadvantages, it is difficult for people to simply apply value maximization. Imagine, for instance, that you are faced with a product buying task in which each option has two attributes and no combination of attributes makes the product perfectly suited to your needs. In such circumstances, when evaluating a particular option, you will necessarily take into consideration not only its features but also the features of all comparable alternatives. Thus, when a new option is added to the original choice set, it may increase or decrease the attractiveness of one or more alternative options in that set, which may change your preferences (Chuang et al., 2012a; Simonson and Tversky, 1992). However, these findings go against the preference invariance hypothesis and have led to the widely studied compromise effect. The compromise effect is theorized based on extensive empirical research on human choice behavior and suggests that an individual’s current context plays a significant role in their decision-making (Chuang et al., 2012b; Noguchi and Stewart, 2014; Payne et al., 1992; Sheng et al., 2005; Simonson and Tversky, 1992). Importantly, the compromise effect tends to explain violations of one of the fundamental assumptions of many rational choice models: that the probability of one option being chosen from an initial choice set cannot be increased by adding a new option (Huber et al., 1982).

Although the compromise effect is an interesting topic of research and has been shown to be robust in numerous studies (e.g., Cheng et al., 2012a,b; Chernev, 2004; Chuang et al., 2012a,b; Dhar et al., 2000; Drolet, 2002; Huber and Puto, 1983), such as those involving cultural differences (Briley et al., 2000), consumer behavior (Cheng et al., 2012a,b; Chuang and Yen, 2007), group decision-making (Dhar et al., 2004; Kivetz et al., 2004b), business-to-business decisions (Dhar et al., 2004; Kivetz et al., 2004b), and technology markets (Kivetz et al., 2004b) in the marketing context, these studies have typically focused on cash payment. Focused on cash payments. Over the past two decades, the frequency with which people use credit cards instead of paper money in payment transactions has increased dramatically (Foster et al., 2013; Shah et al., 2016). In 1999, paper payments (i.e., cash and checks) accounted for approximately 60% of all in-store payments. In 2010, plastic cards (i.e., debit cards, credit cards, and gift cards) were becoming the preferred payment method for most in-store payments, and the proportion of paper payments fell to slightly more than 40% (Shah et al., 2016). The idea that spending behavior is influenced by the payment mechanism is not new. For instance, Hirschman (1979) reported a significant difference in spending amounts when the preferred mode of payment was a credit card, as opposed to other payment methods.

Therefore, a question regarding the compromise effect has emerged. Are there different compromise effects for different payment mechanisms? An examination of the compromise effect in a credit card payment scenario can help to answer these questions. Therefore, this paper aimed to explore the relationship between the compromise effect and payment forms.

### Payment forms and the pain of paying

Rapid changes in the social environment have resulted in significant changes in payment methods. Forty years ago, when people made purchases, they could choose between five payment methods, with cash being the dominant choice. Today, however, there are more than 20 methods of payment, with cashless payments being the dominant choice (Foster et al., 2013). These changes in payment forms have also significantly influenced spending behavior. For example, Feinberg (1986) showed that the amounts of expenses and donations increase significantly when participants are allowed to access credit cards. Prelec and Simester (2001) and Soman and Cheema (2002) also indicated that the use of credit cards leads to an increased willingness to spend and higher spending amounts. The best explanation for this phenomenon is “the pain of paying.” Spending behavior is affected by the pain of paying when parting with one’s money causes discomfort (Prelec and Loewenstein, 1998). The pain caused by the use of cash will leave vivid memory traces, and this pain will be reinforced with each transaction as if it were a punishment. However, credit card purchases only require a signature, so the pain of paying is reduced. Therefore, previous research has indicated that cash payments, as opposed to other forms of payment, cause the greatest pain. Furthermore, extending the notion of payment pain, Chatterlee and Rose (2012) argued that credit card priming draws attention to benefit considerations, whereas cash priming draws attention to costs. In general, people receive benefits when they buy a product or service, but they also have to pay the cost of that product or service. Cash payments have high psychological salience because they involve handing over large amounts of cash, making people realize that they have to pay for a product or service and leading to greater payment pain. Precisely because of the pain of paying, consumers who use cash payments to pay for products or services are more inclined to consider costs and less likely to focus on benefits than those who pay using a credit card. In turn, this process could strengthen the association between cash as a payment method and the costs associated with the products purchased. However, credit card payments help people to decouple costs from benefits and effectively reduce the salience of cost attributes because the painful impact of the payment is not immediate. Indeed, when a person buys a product with a credit card, their purchase is not related to the pain of paying because the payment is only effective a few weeks after the purchase of the product. Nevertheless, consumers’ experiences with the instant gratification of their desire when they pay with a credit card invoke a buy now, pay later mentality (Mendoza and Pracejus, 1997; Shimp and Moody, 2000) and strengthen the association between credit cards and the desired benefits. Repeated credit card purchasing experiences that will eventually lead to immediate gratification appear to make shopping more affordable in terms of cost. From the perspective of payment pain associated with cash payments, Thomas et al. (2011) found that the pain of paying with cash can reduce customers’ desires and encourage them to think more about a purchase, thereby inhibiting the impulse to buy unhealthy foods, such as biscuits, cakes, and pies. However, this pain has little effect on “virtue” products, such as fat-free yogurt and whole wheat bread, because the decision to buy these types of product is based on additional considerations.

Finally, based on prior knowledge regarding the pain of paying with cash, Shah et al. (2016) indicated that compared with people who use less painful payment methods (such as a credit card), those who pay with relatively painful payment methods (such as cash) have an increased post-transaction connection to the purchased product and/or the purchasing organization. Specifically, individuals who use more painful payment forms increase their emotional attachment to products, reduce their commitment to non-selective alternatives, are more likely to publicly express their commitment to the purchasing organization, and are more likely to engage in repeated transactions.

To summarize the discussion so far, the results of previous studies suggest that a high level of pain related to the payment form is associated with both negative decision outcomes (e.g., reduced willingness to spend and reduced willingness to pay) and positive decision outcomes (e.g., enhanced post-purchase satisfaction and brand attachment).

### Compromise effect, payment forms, and the pain of paying

**Figure 1:**
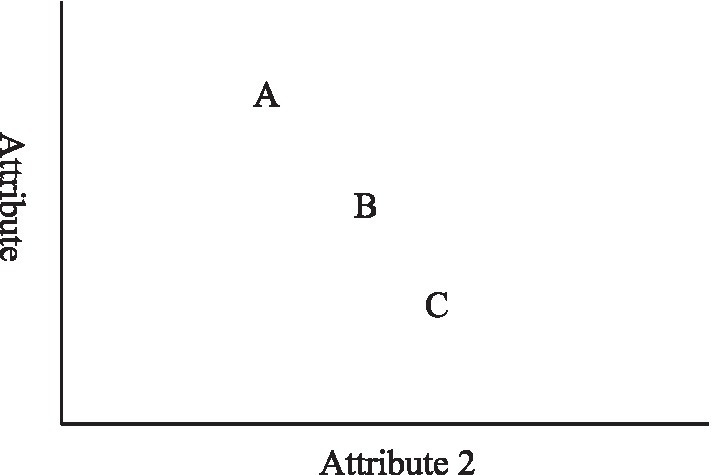
The compromise effect.

The compromise effect—first proposed by Simonson (1989) and extended by Chuang and Yen (2007), Dhar et al. (2000), Drolet (2002), Nowlis and Simonson (2000), and Lin et al. (2006), —posits that a brand’s market share will be greater when it is the middle option in a choice set and will be smaller when it is the extreme option (Simonson and Tversky, 1992). Simonson distinguished the compromise effect from the attraction effect proposed by Huber et al. (1982). Simonson (1989) argued that an option tends to gain market share when it becomes a compromise or middle option within the choice set (see Figure 1). In other words, if a choice set of two options, Options A and B—with Option B being inferior to Option A but less expensive—is expanded to include a third option, Option C (which is inferior to but cheaper than both Options A and B), the relative market share of Option B will increase, as it becomes the compromise option: more economical than Option A, but of better quality than Option C (Chuang et al., 2013; Simonson and Tversky, 1992).

For example, consider that there are initially two cakes in a choice set: a 6-inch cake sold for $10 and an 8-inch cake sold for $15. When a 10-inch cake sold for $20 is added to this choice set, the 8-inch cake becomes the compromise option (the middle option), and the 6-inch and 10-inch cakes become the extreme options. People tend to maximize gains and minimize losses while making decisions (Sheng et al., 2005).

Therefore, in this situation, people will compare the three options and determine that the extreme options have a relative advantage in one of their attributes and a disadvantage in another. In contrast, the attributes of the middle option are between those of the extreme options, so people do not feel that they are losing out in terms of any given attribute (Simonson, 1989; see also Dhar et al., 2000; Kivetz et al., 2004a; Wernerfelt, 1995). Specifically, in a trinary choice set, the extreme options are seen as the riskier options, whereas the middle option is seen as the safe option (Sheng et al., 2005). This provides a basic understanding of the compromise effect. In summary, most decision makers choose the middle option because they believe that it carries less risk and minimizes their expected loss (EL). The value of the middle option is between that of the other alternatives, being neither ideal nor bad (Chernev, 2004).

Sheng et al. (2005) proposed that the EL in a consumer decision is given by:


(1) $$\text{EL}=\sum P_{i}(V_{i}-V_{s})$$

(2) $$\sum P_{i}=1,i=A,B,C$$

where *P_i_* (*i =* A, B, C) is the probability that the *i*th alternative is evaluated as the best choice. V*_i_* is the value of the *i*th alternative, and *V_s_* is the value of the brand chosen by the consumer. Given the uncertainty as to which alternative will be the best choice, it can be assumed that *P*_A_ = *P*_B_ = *P*_C_ = ⅓. Meanwhile, Alternatives A, B, and C are positioned as shown in Figure 1. The shift from the chosen brand is 0, 1, or 2 units of value. Thus, the expected value of each of the options when chosen can be calculated as follows:


$$\begin{array}{l} \text{Choosing}\;A:\text{EL}=P_{A}\;(V_{A}-V_{A})+P_{B}\;(V_{B}-V_{A})+P_{C}\;(V_{C}-V_{A})\\ ={⅓}^{*}\;0+{⅓}^{*}\;1+{⅓}^{*}\;2=1; \end{array}$$

$$\begin{array}{l} \text{Choosing}\;B:\text{EL}=P_{A}\;(V_{A}-V_{B})+P_{B}\;(V_{B}-V_{B})+P_{C}\;(V_{C}-V_{B})\\ ={⅓}^{*}\;1+{⅓}^{*}\;0+{⅓}^{*}\;1=⅔; \end{array}$$

$$\begin{array}{l} \text{Choosing}\;C:\text{EL}=P_{A}\;(V_{A}-V_{C})+P_{B}\;(V_{B}-V_{C})+P_{C}\;(V_{C}-V_{C})\\ ={⅓}^{*}\;2+{⅓}^{*}\;1+{⅓}^{*}\;0=1. \end{array}$$

The results demonstrate that choosing Option B can effectively minimize the EL. However, Chuang et al. (2013) found that self-confident people are less likely to expect losses when making decisions and take more risks, so they are less likely to choose the safe (or middle) option in a large choice set. In contrast, Chuang et al. (2012a) found that incomplete information reduces the customer’s uncertainty and increases their sense of loss, thus encouraging them to choose the middle option.

Increasing purchase quantity often reduces the compromise effect due to variety-seeking and the application of balance heuristics. This phenomenon may also extend to payment choice contexts. When consumers are faced with repeated or multiple transactions, they may avoid relying on a single “compromise” payment method. Instead, they tend to distribute their payment decisions across different tools to achieve psychological balance and alleviate the pain of paying (Cheng et al., 2012a). This cognitive tendency may similarly influence payment-related decisions, especially when multiple payment methods are available, each with its own advantages and disadvantages.

Furthermore, from an interpersonal perspective, when consumers make payment-related decisions on behalf of others, particularly for individuals with whom they have a distant relationship, they may be more inclined to choose a compromise or safer option. This tendency not only affects product selection but also extends to payment method choice, offering deeper insight into consumer payment behavior in social contexts (Chang et al., 2012).

Based on the discussion above, the pain of paying with cash will draw more attention to the cost or disadvantages of a product, leading people to increase the salience and weight of a product’s disadvantages. Thus, when faced with a choice set of three comparable alternatives, none of which is clearly better, the middle option is seen as minimizing the EL. Because cash payments increase customers’ sense of loss, they are more likely to choose the middle option. In contrast, credit card payments are not associated with the pain of paying. Indeed, people who pay with a credit card focus more on the benefits of the product under consideration, which reduces their sense of loss, so they are less likely to choose the middle option. Based on the above discussion, we propose the following hypotheses

*H1*: The compromise effect is weaker when consumers pay with a credit card than when they pay with cash.

*H2*: The strong compromise effect in a cash payment situation (vs. credit card payment) is mediated by the pain of paying.

### Tightwads and spendthrifts as moderators

People inherently experience different levels of payment pain. Some people seem to be particularly sensitive to the pain of paying, whereas others seem to be immune. The pain of paying can affect a consumer’s behavior differently depending on their ideal consumption patterns. Rick (2018) identified two types of people with different sensitivities to the pain of paying: tightwads and spendthrifts.

Tightwads, who experience the pain of paying intensely, may end up spending less than they would like. That is, in many cases, they think that they should buy something, but their pain prevents them from acting on that desire. In contrast, spendthrifts, who experience a low level of payment pain, may end up spending more than they would like. As a result, tightwads are particularly sensitive to marketing contexts that make spending less painful (Rick, 2018).

Payment forms significantly influence consumer decision-making beyond the transaction itself. For instance, Huang et al. (2025) found that consumers exhibit different preferences for promotional offers depending on whether they pay with cash or credit cards. Specifically, cash users tend to prefer price discounts when prompted by a calculating mindset, while credit card users are more drawn to bonus packs when guided by emotional valuation. Moreover, this relationship is moderated by individual traits such as self-construal (independent vs. interdependent) and sensitivity to payment pain (tightwads vs. spendthrifts). These findings highlight the psychological mechanisms linking payment choice to value perception and offer structure, providing important implications for understanding consumer behavior in increasingly cashless contexts.

In general, tightwads spend less than spendthrifts, although there are contexts that mitigate the influence of differences in spending between tightwads and spendthrifts. For example, in the study by Rick (2018), for “vice” products, the differences in spending between tightwads and spendthrifts were smallest when the participants paid with a credit card (the relatively less painful payment method). Frederick et al. (2009) also found the smallest difference between tightwads and spendthrifts. Spendthrifts were significantly less likely to choose the more expensive stereo when the salience of opportunity costs was highlighted than when it was not. In contrast, tightwads were not significantly influenced by the salience of opportunity costs.

Taken together, the results of these studies suggest that individuals differ in their chronic propensity to experience the pain of paying. Compared with spendthrifts, tightwads are more sensitive to the pain of paying so when paying in cash, they will pay more attention to the disadvantages of a choice set. Our conceptualization suggests that the effect of cash payments on the compromise effect will be stronger for tightwads than for spendthrifts because the former will experience the pain of paying more. Based on the above discussion, we propose the following hypotheses

*H3*: The effect of cash payments on the compromise effect is stronger for tightwads than for spendthrifts.

## Materials and methods

We conduct three studies to test the influence of payments on the compromise effect. In study 1, we directly manipulate cash/credit card payments situations to provide evidence to support H1. We design study 2 to provide greater insights into the underlying mechanism (the pain of paying.) and excluded alternative explanations such as differences in price, product category, and attribute importance. Finally, in study 3, we explore the impact of cash payments on the compromise effect was stronger among tightwads than among spendthrifts.

### Study 1: payment forms and the compromise effect

In Study 1, we manipulated the payment forms in a laboratory experiment, keeping all other elements constant. If paying with a credit card reduces the compromise effect, then we can attribute the observed effect exclusively to the payment form. We predict that when consumers use a credit card to pay, the compromise effect is weaker than when they use cash to pay.

#### Participants and design

Study 1 was designed to test the influence of payment forms on the compromise effect, based on the assumption that the compromise effect is weaker in credit card payments than in cash payments. Three hundred and sixty-four graduate and undergraduate students (52.2% female; 47.8% male) agreed voluntarily to participate in the study in exchange for course credits. Their ages ranged from 19 to 69 years (average age = 31.1 years).

Study 1 used a 2 (payment: credit card or cash) × 2 (compromise choice set: binary or trinary) between-subjects design. The participants were randomly assigned to one of four groups: binary set based on cash payment, binary set based on credit card payment, trinary set based on cash payment, and trinary set based on credit card payment. The choice set and procedure for Study 1 were adapted from Simonson (1989). The participants were asked to imagine that they had decided to buy a USB flash drive.

#### Manipulation of the payments

The manipulation of the cash and credit card payment conditions followed the study by Shah et al. (2016). In the cash payment condition, the participants were told that they could only purchase the USB flash drive in cash. In the credit card payment condition, the participants were told that they could only purchase the USB flash drive with a credit card, debit card, or a prepaid university card commonly used on campus.

#### Manipulation of the compromise effect

The stimulus product adopted in Study 1 was similar to those used in prior studies on the compromise effect (Chuang et al., 2012a; Lee et al., 2017; Sheng et al., 2005; Simonson, 1989). The product in each given choice set (i.e., binary or trinary) had two attributes, which were typical of those highlighted in the marketing materials for this product at the time of data collection. In the binary choice set, as shown in Table 1, the two attributes of the product included one advantage and one disadvantage vis-à-vis the other option in that set. As discussed in the above literature review on the compromise effect, adding a new option to a binary choice set can change the likelihood of choosing one of the original two options. In particular, Study 1 was intended to determine, for each choice task, how the relative probability of a participant favoring Option A or Option B changed after Option C was added to the original binary set.

**Table 1:** Product categories and attributes.

Product	Brand	Attribute 1	Attribute 2
USB flash drive		Price ($)	Storage capacity (GB)
	A	60	512
	B	40	256
	C	20	128
Box of chocolates ($30)		Taste	Number of flavors
	A	Excellent	4
	B	Delicious	7
	C	Good	10
Thermos bottle ($80)		Keeps liquids hot or cold (hours)	Capacity (L)
	A	24	1
	B	16	1.5
	C	8	2

#### Measure of the compromise effect

In general, the compromise effect was assessed by comparing the participants’ overall preference or “market share” for the two options in the binary sets and the two original options in the trinary sets. Following Chuang and Yen (2007), P(B; A) referred to the share of Option B from a binary choice set {A, B}, and P_c_(B; A) referred to the share of Option B relative to Options A and C in the trinary set {A, B, C}. As noted in the literature review, the compromise effect can be said to occur when the relative preference for Option B is higher in the trinary set than in the binary set (Nagpal and Krishnamurthy, 2007). The compromise effect was therefore computed as the change ΔP in the share of Option B relative to Option A after adding Option C to the main set {A, B}, that is, ΔP = [P_c_ (B; A) - P (B; A)] (Simonson and Tversky, 1992). Accordingly, the presence of the compromise effect is confirmed when ΔP is positive (see Chuang et al., 2012a).

#### Results

In Study 1, H1 predicts that the compromise effect would be weaker when paying with a credit card. H1 will be confirmed when the interaction effect between the choice set and payment form is significant (cash vs. credit card), and when ΔP_cash_ is significantly greater than ΔP_credit_. To examine the significance of the difference, we calculated a 2 (choice set) × 2 (cash payment) log-linear model using SPSS 13.0 software. A log-linear analysis is a statistical technique for analyzing data when both the independent and dependent variables are categorical or nominal (Tansey et al., 1996). As predicted, the interaction between the payment (cash vs. credit card) and choice set was significant (χ^2^ = 4.01; *p* < 0.05). The compromise effect was weaker for credit card payments because the share of the target option decreased from 51% in the binary set to 48% in the trinary set, resulting in a compromise effect of −3% (χ^2^ = 0.067; *p* > 0.5). In contrast, in the cash payment condition, the compromise effect increased from 48% in the binary set to 69% in the trinary set, with a compromise effect of 21% (χ^2^ = 6.6, *p* < 0.01). These results support H1, which states that paying with a credit card can reduce the compromise effect.

### Study 2: ruling out alternative explanations in an incentive-compatible design

Study 1 showed that the compromise effect was greater in the cash payment condition than in the credit card payment condition. However, three limitations were observed in the effect of payment forms on the compromise effect. First, the stimulus in Study 1 had a more utilitarian aspect. Kim and Kim (2014) indicated that utilitarian products are more likely than hedonic products to encourage customers to choose the middle option, which would affect the likelihood of the participants choosing the middle option in Study 1. Thus, the observed impact of payment forms on the compromise effect could be attributed to the product type. To rule out alternative explanations regarding the effect of payment forms on the compromise effect, in Study 2, we asked the participants to choose between two types of products (i.e., a box of chocolates or a thermos bottle). The box of chocolates (a hedonic product) differed in terms of the taste and number of flavors, and the thermos bottle differed in terms of the capacity (L) and ability to keep liquids hot or cold (for details, please see Table 1). The participants were exposed to three options based on different experimental conditions, and the order of the products was also mixed.

Second, prior studies have shown that an asymmetric weighting of attributes between price and quality reduces the probability that a consumer will select the compromise option (Sheng et al., 2005; Simonson and Tversky, 1992). This suggests that consumers are less likely to choose the middle option when paying with a credit card, potentially due to the price attributes of the products. Additionally, a study on the impact of self-confidence on the compromise effect analyzed consumer behavior in a three-option selection scenario using high-priced laptops. The findings confirmed that consumers consistently gravitate toward the compromise option, aligning with our observations in studies involving lower-priced products (Chuang et al., 2013). From the perspective of price precision, when prices are presented in exact numerical values, the complexity of decision-making increases, which may weaken the compromise effect. In other words, the more precise and complex the pricing, the more likely consumers are to engage in price-driven decision-making (Cui et al., 2021). However, the compromise effect remains consistent across different price levels. Premium pricing further amplifies decision complexity, compelling consumers to conduct extensive information gathering before making a purchase (Lee et al., 2017). Therefore, we excluded price as an attribute from the two product categories in Study 2. Third, attribute weighting could explain the magnitude of the compromise effect. Specifically, the compromise effect may be stronger when the two attributes are equally important. Therefore, in Study 2, the lower probability of choosing the middle option when paying with a credit card could be driven by the asymmetric importance of the two product attributes (thermos bottle: keeps liquids hot or cold and capacity; box of chocolates: taste and number of flavors). Thus, to rule out this alternative explanation, I conducted a pretest with 38 participants by asking them about “How important do you think capacity (keeps liquids hot or cold) is to you when you purchase thermos bottle?” and “How important do you think taste (number of flavors) is to you when you purchase box of chocolates?” to verify the equal importance of the two product attributes: keeps liquids hot or cold and capacity (importance: *M* = 4.94 vs. *M* = 4.97, *t* = 0.167, *p* > 0.10) for the thermos bottle, and taste and number of flavors (importance: *M* = 5.20 vs. *M* = 5, *t* = 1.05, *p* > 0.10) for the box of chocolates. If the choice based on different attribute weights is a key mechanism underlying the compromise effect, I should observe a significant compromise effect of the products with equally weighted attributes.

#### Participants and design

In Study 2, we used an incentive-compatible design to elicit real customer preferences and understand real decision situations by asking the participants to indicate their preferred product along with the likelihood of acquiring said preferred product (Curley et al., 1986; Liu and Chou, 2018).

Four hundred and twenty graduate and undergraduate students (50.7% female; 49.3% male, average age = 32.99 years) participated in the experiment. Those who participated in Study 1 were not allowed to participate in this study to avoid unnecessary repeat testing. The 420 participants were randomly assigned to one of the cells in a 2 (payment: cash vs. credit card) × 2 (compromise effect set: binary vs. trinary) between-subjects design. The manipulation of the factors (payment and compromise effect) was the same as in Study 1. Two product categories (thermos bottle and box of chocolates) were used in Study 2, as shown in Table 1. Once the participants completed the choice task, they were asked to record their feelings about the pain of paying using a 7-point scale that ranged from (*How painful was paying for the*) thermos bottles (chocolates) to (*How painful was giving up your money*) (*r* = 0.79) (Shah et al., 2016). Finally, based on incentive-compatible theory, the participants will have a chance to obtain the experimental products (three thermos bottles and three boxes of chocolates).

#### Results

The measurement and analysis techniques used to determine the compromise effect were the same as in Study 1. We used a 2 (compromise choice set: binary vs. trinary) × 2 (payment: cash vs. credit card) log-linear model and SPSS 20.0 software. The data analysis confirmed our prediction and showed a salient interaction between the payment form and the compromise effect (thermos bottle: χ^2^ = 5.2, *p* < 0.05; box of chocolates: χ^2^ = 9, *p* < 0.005). For the participants in the cash payment condition, the market share of Option B in the compromise choice set increased by 16% for the thermos bottle (from 48% in the binary set to 64% in the trinary set; χ^2^ = 4.36, *p* < 0.05) and by 26% for the box of chocolates (from 45% in the binary set to 71% in the trinary set; χ^2^ = 12.22, *p* < 0.001). However, the market share of Option B fell by −8% for the thermos bottle (from 55% in the binary set to 47% in the trinary set; χ^2^ = 1.28, *p >*0.1) and by −5% for the box of chocolates (from 55% in the binary set to 50% in the trinary set; χ^2^ = 0.478, *p >*0.1) among the participants in the credit card payment condition. These results support H1.

#### Mediating effect through the pain of paying

H2 predicts that the pain of paying mediates the effect of cash/credit card payments on the compromise effect. According to our theoretical reasoning, the pain of paying should mediate the effect of cash/credit card payments on the compromise effect. We performed a mediation analysis using the method proposed by Hayes (2013) (PROCESS Model 4) to test our prediction. The mediator of the compromise effect (the pain of paying) was adopted from Kim and Kim (2014). The participants’ choice was modeled as a binary variable, with 1 indicating that the compromise option was chosen and 0 indicating that another option was chosen. The payment type was also treated as a binary variable, with 0 indicating a cash payment and 1 indicating a credit card payment. We first analyzed the participants’ feelings of pain when paying with cash or with a credit card. The results showed the participants felt more pain in the cash payment condition, which was consistent with our expectations (thermos bottle: *M*_cash_ = 4.76 vs. *M*_credit_ = 3.33, *t* = 2.16, *p* < 0.05; box of chocolates: *M*_cash_ = 3.69 vs. *M*_credit_ = 3.41, *t* = 194, *p* < 0.05). This finding supports our theory that paying with a credit card is less painful than paying with cash and reduces the compromise effect. Furthermore, the results showed that the indirect effect of cash/credit card payments on the compromise effect was mediated by the pain of paying (thermos bottle: indirect effect = −0.377, *SE* = −0.85, 95% CI [−0.774, −0.052]; box of chocolates: indirect effect = −0.2015, *SE* = −0.1157, 95% CI [−0.485, −0.0144]). In conclusion, the effect of a credit card payment was significant and reduced the compromise effect. Indeed, credit card payments are likely to be less painful for customers, prompting them to focus on the benefits when evaluating a product and thereby reducing their likelihood of selecting the middle option. This result supports H1, which postulates that the credit card payments reduce the compromise effect.

### Study 3: tightwads and spendthrifts as a moderator

As previously mentioned, Rick (2018) argued that individuals have different long-term sensitivities to payment pain. Compared with spendthrifts, tightwads experience more pain when spending money. H3 predicts that the effect of cash payments on the compromise effect is greater for tightwads than for spendthrifts because the former will experience more pain. Study 3 tested this hypothesis.

#### Participants and design

Four hundred and eighty participants (213 men and 267 women aged 18–65 years; mean age = 32.4 years) were recruited from MTurk to participate in this study. All participants in this shopping scenario were randomly assigned to two payment conditions (cash vs. credit card) and the two choice set conditions (binary vs. trinary). The manipulation of the payment types and the compromise effect, the stimuli, and the procedure were the same as in Study 2.

After completing their choice task, the participants were asked to complete the tightwad–spendthrift (TW-ST) scale developed by Rick (2018) to measure individual differences in the pain of paying. The scale uses four items to measure the spending habits of the participants when shopping. The scale items examine whether consumers have difficulty controlling their spending (spendthrifts) or whether they have difficulty forcing themselves to spend (tightwads). A higher score on this scale indicates that the participant feels less pain when paying and is a spendthrifts. Before completing this scale, the participants were clearly told that the questions on the scale were related to their usual consumer behavior.

#### Results

To examine H3, a log-linear analysis was conducted. H3 predicts that the effect of cash payments on the compromise effect is stronger for tightwads than for spendthrifts. The data analysis supported our prediction and revealed a three-way significant interaction among tightwads/spendthrifts, payment forms, and the compromise effect [thermos bottle: χ^2^ (1) = 3.992, *p* < 0.05; box of chocolates: χ^2^ (1) = 5.44; *p* < 0.05]. To determine the direction of this effect, the results show a significant interaction between the payment form and the compromise effect among spendthrifts [thermos bottle: χ^2^ (1) = 4.09; *p* < 0.05; box of chocolates: χ^2^ (1) = 4.34; *p* < 0.05]. However, among tightwads, we found no significant interaction [thermos bottle: χ^2^ (1) = 0.63; *p* > 0.1; box of chocolates: χ^2^ (1) = 1.43; *p* > 0.1]. These results support our inference about the moderating role of tightwads/spendthrifts on the interaction between payment forms and the compromise effect. In addition, for spendthrifts, the preference for Option B increased by 20%, in the cash payment condition and decreased by −9% in the credit card payment condition, a percentage difference of 29%. For tightwads, the preference for Option B decreased by −11%. Thus, H3 was supported.

#### Pain of paying

We conducted a *t*-test to compare the payment pain experienced between tightwads and spendthrifts. The results showed that tightwads felt more pain than spendthrifts (*M*_tightwads_ = 5.41 vs. *M*_spendthrifts_ = 2.57, *t* = 23.27, *p* < 0.001), which is consistent with our expectations. Specifically, tightwads are more sensitive to pain than spendthrifts when they spend money, and this influences the effect of the payment form on the compromise effect.

## Discussion

Studies have increasingly evaluated how the compromise effect influences consumer choices. The compromise effect has traditionally been discussed in terms of its underlying cognitive processes (Mishra et al., 1993; Ratneshwar et al., 1987; Simonson, 1989), and recent studies have focused on individual decision makers and the effect of social context (Briley et al., 2000; Chuang and Yen, 2007; Nowlis and Simonson, 2000; Sheng et al., 2005). The literature has also pointed out that cash–credit differences exist in many situations depending on the context (Beach and Mitchell, 1987; Heath, 1996; Kray and Gonzalez, 1999). Therefore, this paper adds to the literature on the compromise effect by examining how cash and credit card payments affect this effect by using different product categories (e.g., USB flash drive, box of chocolates, and thermos bottle) and excluding alternative explanations such as differences in price and product category. The results of the three empirical studies provide converging evidence showing that the compromise effect is weaker when paying with a credit card than when paying with cash.

Study 1 examined whether consumers are more likely to choose the middle option in a choice set when they are asked to pay with cash than with a credit card. The results are consistent with our prediction; that is, the participants were more likely to choose the middle option in the cash payment scenario than in the credit card payment scenario. To better understand the effect of the payment form on the compromise effect, we further analyzed whether the pain of paying mediated the compromise effect. Consistent with our prediction, the results showed that paying with cash resulted in more pain when spending and a greater compromise effect, compared to paying with a credit card. These results support the theory that the effect of the payment form on the compromise effect is due more to the pain of paying than to the types of products and the weights of their attributes. These results imply that people who feel more pain when spending tend to choose what they really like, rather than the compromise alternative.

Study 2 used different product categories and provided equal weighting to the product attributes, and the results showed that the participants had a weaker compromise effect when paying with a credit card than when paying with cash. This allowed us to successfully rule out alternative explanations. The results further support our main results in the context of the effect of the payment form on the compromise effect. Study 3 investigated the moderating effects of individual differences in sensitivity to the pain of paying on the compromise effect. The results showed that the cash payment on compromise effect was stronger for tightwads than for spendthrifts. That is, tightwads feel more pain when spending and tend to focus more on the disadvantages and less on the advantages of a choice set, so they tend to choose the compromise alternative rather than what they really like. Together, these studies convincingly demonstrate that the pain of paying is the mechanism behind the compromise effect of cash payments.

This study contributes to the literature on the compromise effect (Cheng et al., 2012a,b; Chernev, 2004; Chuang et al., 2012a,b; Dhar et al., 2000; Drolet, 2002; Huber and Puto, 1983) by demonstrating that the compromise effect is a function of cash payments versus credit card payments. Unlike most studies of the compromise effect, which focused on paying with cash, the three studies presented in this article illustrate how paying with a credit card moderates the impact of the compromise effect. This study extends the notion of the compromise effect to the area of payment types, thus highlighting the role of payment pain. It draws on prior research on the effect of credit card payments on spending, which has typically shown that customers feel more pain of paying and place more weight on the advantages of product attributes, thereby reducing the compromise effect. In this broader context, our paper demonstrates that the compromise effect is a function of payment forms induced by the pain of paying.

The findings of this paper can facilitate the assessment of the psychological underpinnings of payment pain and cash–credit differences, and can help to identify the factors at play in trade-off strategies between cash and credit card payments. We believe that a better understanding of these mechanisms can guide marketers interested in increasing sales by providing a more accurate understanding of consumer differences and how they relate to payment forms. In addition, our findings shed light on the layout of product preferences for marketers by identifying the factors that cause consumers to make decisions that are consistent or inconsistent with their original preferences and encourage them to buy the promoted product with a designated credit card payment.

### Limitations and directions for future research

This paper has two limitations that should be acknowledged. First, although this paper identified the potential influence of payment forms (cash or credit card) on the compromise effect during decision-making, this finding does not mean that this influence is limited to this factor. Thus, other factors not considered here may also emerge as important as the body of literature grows. In addition, our sample sizes in Study 1 and Study 2 were relatively small and homogenous (i.e., university students). Future research should focus on other populations to verify our results. Second, although the experiments were similar to those carried out in previous studies and seemed acceptable to the participants, the conditions were imaginary. Therefore, the level of engagement of the participants in the manipulated scenarios and the quality of their answers are unknown. Third, only two product categories were used in the experiments, which may limit the external validity of the findings. An important question that should be discussed in future research concerns the moderating effects of other individual differences on the relationship between payment forms and the compromise effect. Fourth, regarding product pricing, this study focused solely on low-priced products. As a result, the impact of the compromise effect in high-priced product contexts remains unknown. Higher price points may introduce additional decision-making complexities, potentially influencing consumer behavior differently. Future research should explore whether the compromise effect manifests similarly when premium pricing is involved.

## Data Availability

The original contributions presented in the study are included in the article/supplementary material, further inquiries can be directed to the corresponding author/s.
